# Structural Elements Recognized by Abacavir-Induced T Cells

**DOI:** 10.3390/ijms18071464

**Published:** 2017-07-07

**Authors:** Daniel Yerly, Yuri Andreiw Pompeu, Ryan J. Schutte, Klara. K. Eriksson, Anette Strhyn, Austin. W. Bracey, Soren Buus, David A. Ostrov

**Affiliations:** 1 Department of Rheumatology, Immunology and Allergology, University Hospital of Bern, 3010 Bern, Switzerland; daniel.yerly@allergy.unibe.ch (D.Y.); klara.eriksson@allergy.unibe.ch (K.K.E.); 2 Harvard Medical School, Cambridge, MA 02138, USA; Yuri_Pompeu@hms.harvard.edu; 3 Department of Pathology, Immunology and Laboratory Medicine, University of Florida College of Medicine, Gainesville, FL 32610, USA; rschutte@ufl.edu (R.J.S.); bracey.a@ufl.edu (A.W.B.); 4 Department of Microbiology and Immunology, University of Copenhagen, 1165 København, Denmark; astryhn@sund.ku.dk (A.S.); sbuus@sund.ku.dk (S.B.)

**Keywords:** drug hypersensitivity, Human Leukocyte Antigen, crystallography

## Abstract

Adverse drug reactions are one of the leading causes of morbidity and mortality in health care worldwide. Human leukocyte antigen (HLA) alleles have been strongly associated with drug hypersensitivities, and the causative drugs have been shown to stimulate specific T cells at the sites of autoimmune destruction. The structural elements recognized by drug-specific T cell receptors (TCRs) in vivo are poorly defined. Drug-stimulated T cells express TCRs specific for peptide/HLA complexes, but the characteristics of peptides (sequence, or endogenous or exogenous origin) presented in the context of small molecule drugs are not well studied. Using HLA-B*57:01 mediated hypersensitivity to abacavir as a model system, this study examines structural similarities of HLA presented peptides recognized by drug-specific TCRs. Using the crystal structure of HLA-B*57:01 complexed with abacavir and an immunogenic self peptide, VTTDIQVKV SPT5a 976–984, peptide side chains exhibiting flexibility and solvent exposure were identified as potential drug-specific T cell recognition motifs. Viral sequences with structural motifs similar to the immunogenic self peptide were identified. Abacavir-specific T cell clones were used to determine if virus peptides presented in the context of abacavir stimulate T cell responsiveness. An abacavir-specific T cell clone was stimulated by VTQQAQVRL, corresponding to HSV1/2 230–238, in the context of HLA-B*57:01. These data suggest the T cell polyclonal response to abacavir consists of multiple subsets, including T cells that recognize self peptide/HLA-B*57:01 complexes and crossreact with viral peptide/HLA-B*57:01 complexes due to similarity in TCR contact residues.

## 1. Introduction

Although there are relationships between viral infections and immune-mediated adverse reactions, the mechanistic roles of the viruses are complex and incompletely defined [1,2]. Cytotoxic T lymphocytes specific for viral epitopes have been shown to be expanded in patients with drug hypersensitivity reactions [3], suggesting that drugs stimulate pathogenic T cells previously primed by virus exposure (a cross-reaction/molecular mimicry mechanism) [4]. Offending drugs have been shown to stimulate virus reactivation and expression of viral proteins (directly or indirectly) [3], thus resulting in increased presentation of viral epitopes and expansion of virus specific T cells that may promote hypersensitivity.

Examples of relationships between viral infections and increased risk of developing immune-mediated adverse reactions suggest that multiple viruses may be involved. In human immunodeficiency virus (HIV) patients, severe adverse reactions to co-trimoxazole are significantly more frequent compared to uninfected patients [5,6]. Human herpes virus 6 (HHV-6) is associated with drug reaction with eosinophilia and systemic symptoms (DRESS) induced by carbamazepine, teicoplanin and vancomycin [7]. Cytomegalovirus (CMV) is associated with tribenoside-induced hypersensitivity syndrome [8]. These data suggest that virus infection influences the initiation or perpetuation of drug hypersensitivity.

One hypothesis to explain the role of viruses in drug hypersensitivity is that cytotoxic T cells specific for viral antigens cross-react with self peptides complexed to drugs and HLA molecules [9]. A consequence of this would include the generation of T cells specific to anatomical sites where specific self and/or viral antigens are expressed. Advancements in understanding the structural mechanisms responsible for immunopathogenesis of abacavir hypersensitivity syndrome, an autoimmune response following treatment with the HIV drug strongly associated with HLA-B*57:01 [10,11], allows the abacavir T cell recognition system to serve as a model for assessing which peptide antigens are presented by HLA-B*57:01 [1].

To understand the structural elements recognized by drug-specific T cells, we mapped solvent accessible elements of an immunogenic self peptide complexed with abacavir and HLA-B*57:01. Potentially cross-reactive viral epitopes were then identified by sequence homology to the self peptide. Finally, we used a TCR transfection system to determine if identified homologous viral peptides could be recognized by drug reactive T cells.

## 2. Methods

### 2.1. X-ray Crystallography and Structural Analysis

Refolded β_2_-microglobulin, HLA-B*57:01, self peptide VTTDIQVKV, and abacavir formed crystals at 4 mg/mL in 0.17 M sodium acetate trihydrate, 0.1 M sodium cacodylate (pH 6.5), 20% PEG 8000, and 15% glycerol. These crystals belonged to space group P2_1_ and contained two HLA heterodimers (heavy chain HLA-B*57:01 and light chain β2-microglobulin) in the asymmetric unit. The phasing was done by molecular replacement using PDB 3UPR as a model (HLA-B*57:01 complexed to abacavir and a synthetic peptide, HSITYLLPV). HKL2000 [12] was used to index the data and generate reflection files, which were used for phasing and refinement in PHENIX [13]. The best crystals exhibited unit cell dimensions a = 45.1 Å, b = 132.6 Å, c = 88.0 Å, α = 90°, β = 105.0°, and γ = 90°. The structure was refined to an R value of 18.2% and an R_free_ value of 24.2% using X-ray diffraction data to 2.0 Å (PDB code 5U98).

The conformations of peptides complexed to abacavir and HLA-B*57:01 were compared using peptides from PDB 5U98; VTTDIQVKV, 3VRI; RVAQLEQVYI, 3VRJ; LTTKLTNTNI, 3UPR; HSITYLLPV. PDB files were superimposed with SSM in Coot (Crystallographic Object-Oriented Toolkit) using 5U98 as a reference.

### 2.2. TCR-Transfectant Stimulation Assay

JRT3-CD8 cells expressing the abacavir-specific TCR 2D, UL3L or BeS-B7 TCR were cocultured in 200 μL of medium with 721.221 cells expressing the HLA-B*57:01 molecule at 1:2 ratio at 37 °C. Abacavir and/or given peptides were added to the cocultures with the indicated concentrations. After 16 h, cells were stained with anti-human CD3 (PerCp-Cy5.5, Biolegend, San Diego, CA, USA) and anti-human CD69 (APC, Biolegend) and analyzed by flow cytometry on a FACS-Canto-I (BD-Biosciences, San Jose, CA, USA). Increase of CD69 expression was monitored in 10,000 CD3^+^ events. Experiments were repeated 3 times and results are given as mean +/− standard deviation.

## 3. Results

### 3.1. Defining TCR Contact Residues in a Self Peptide Complexed to Abacavir and HLA-B*57:01

**Figure 1:**
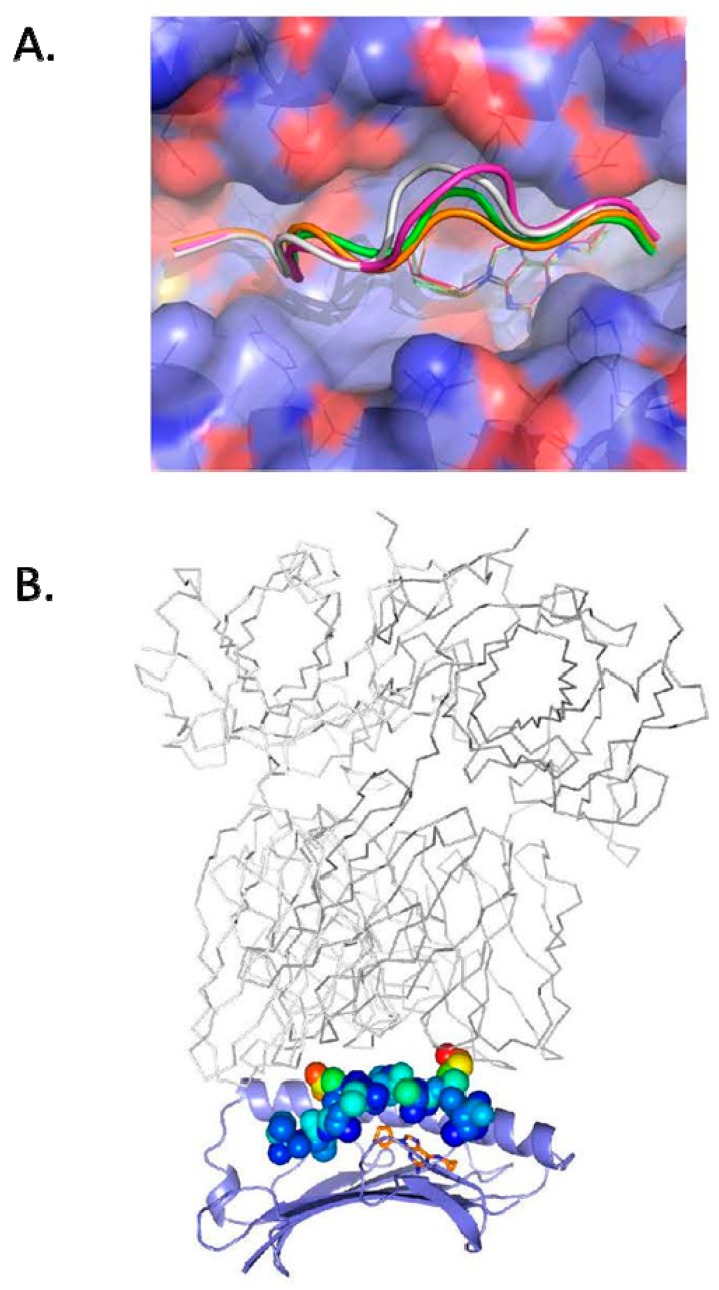
The crystal structure of self peptide VTTDIQVKV SPT5a 976–984 complexed to abacavir and HLA-B*57:01 reveals solvent accessible side chains available for recognition by T cells. (**A**): abacavir is shown in the crystal structure of VTTDIQVKV SPT5a 976–984/HLA-B*57:01 as gold sticks. Binding of abacavir was similar to solved structures of other peptides complexed with abacavir and HLA-B*57:01: PDB code 5U98, gold for carbon, 3VRI, magenta, 3VRJ, white, 3UPR, green. The molecular surface of HLA-B*57:01 is shown as violet for carbon, blue for nitrogen, red for oxygen. (**B**): the crystal structure of VTTDIQVKV SPT5a 976–984 complexed to abacavir and HLA-B*57:01 is shown with the peptide as spheres colored by B factor. P4D and P8K exhibiting the greatest degree of flexibility and solvent exposure. The HLA-B restricted TCR (gray lines) from 2NX5 was superimposed on HLA-B*57:01 to show a conventional binding mode of TCR with respect to peptide and HLA.

Solved structures of peptides bound to HLA-B*57:01 in the presence of abacavir illustrate that the drug forms peptide/HLA-B*57:01 interactions by direct H bonds and van der Waals contacts with both elements. Ordered water molecules mediate indirect contacts between abacavir, peptide and HLA-B*57:01. These data demonstrate that abacavir is buried and not directly accessible to the TCR (Figure 1). Since the structures of peptides complexed to abacavir and HLA-B*57:01 assume conformations similar to peptides bound to other class I HLA molecules, abacavir reactive T cells presumably recognize a surface motif comprised of peptide and HLA-B*57:01. Conventional T cell recognition involves contact between TCR CDR loops and solvent exposed side chains of HLA bound peptides. We asked which peptide elements presented by HLA-B*57:01 are solvent-exposed in the context of abacavir, to elucidate the structural elements recognized by drug-induced T cells. We solved the structure of a peptide, corresponding to human transcription elongation factor SPT5 isoform A, VTTDIQVKV, 976–984, complexed to abacavir and HLA-B*57:01. SPT5a 976–984 was previously identified by characterization of HLA-B*57:01 bound peptides from cells treated with abacavir. SPT5a 976–984 binds HLA-B*57:01 with high affinity in the presence of abacavir (K_d_ approximately 0.2 nM, compared to 7.3 μM in the absence of abacavir) and stimulates T cells from patients with abacavir hypersensitivity syndrome.

X-ray data was refined to a resolution limit of 2.0 Å, and the structure was compared with other solved structures of abacavir bound to HLA-B*57:01 and peptide, Figure 1A. When complexed with 4 different peptides and HLA-B*57:01, abacavir adopts the same docking conformation with the cyclopropyl group occupying the F pocket and abacavir N atoms forming hydrogen bonds with Ser116 and Asp114, residues unique to HLA-B*57:01.

The solved structure demonstrates that V**T**TDIQVK**V** SPT5a 976–984 has the expected anchor residues at P2 (Thr) and P9 (Val) with side chains buried and inaccessible to solvent. The peptide side chains in the core of the peptide that are most solvent exposed (and available for TCR recognition) are P4 (Asp, 105.4 Å^2^) and P8 (Lys, 130.4 Å^2^) in VTT***D***IQV***K***V SPT5a 976–984, Figure 1B.

To understand which peptide side chains may interact with TCRs docking in a conventional binding mode, we superimposed the solved crystal structure of a complex between an HLA-B restricted TCR, EBV peptide EPLPQGQLTAY, and HLA-B*35:01 (PDB 2NX5) on SPT5 isoform A, VTTDIQVKV, 976–984, complexed to abacavir and HLA-B*57:01. These data suggest that P4 (Asp) and P8 (Lys) in the self peptide SPT5a 976–984 have the potential to contact the TCR directly. The side chain atoms at P4 and P8 of VTT***D***IQV***K***V SPT5a exhibit the highest B factors in the peptide, indicating the flexibility of the solvent-exposed side chains free to engage the TCR. These data suggest a mechanism in which drug-responsive T cells recognize a complex of abacavir bound to both peptide and the HLA, where the drug is sequestered in the antigen-binding cleft and TCRs contact HLA-B*57:01 and solvent exposed self peptide side chains.

### 3.2. Identification of Viral Peptides Similar to a Drug-Restricted Self Epitope

BLAST was used to search non-redundant protein sequences from the viridae set to identify viral sequences similar to self peptide VTTDIQVKV [14]. As shown in Table 1, sequences from HIV and Human Simplex Viruses were similar to the VTTDIQVKV self peptide, ranging from 44.4 to 77.8% identity [15,16,17,18]. These viral peptides exhibited the motif for peptides capable of binding HLA-B*57:01 in the presence of abacavir: nonamers with a preference for threonine at P2 and valine and leucine at P9 anchors. The most similar viral sequence, VTTNIQTKV HIV-1 153–161, corresponded to the gp120 Env V2 loop (sequence numbering from viral strain HXB2, GenBank Accession Number K03455). Since these viral peptides would be expected to bind HLA-B*57:01 in the presence of abacavir, the peptides with solvent-exposed TCR contact residues at positions P4 and P8, similar to VTT***D***IQV***K***V SPT5a 976–984, would be expected to stimulate drug-reactive T cells.

### 3.3. Abacavir Reactive T Cells Recognize a Herpes Simplex Peptide in the Context of Drug and HLA-B*57:01

**Figure 2:**
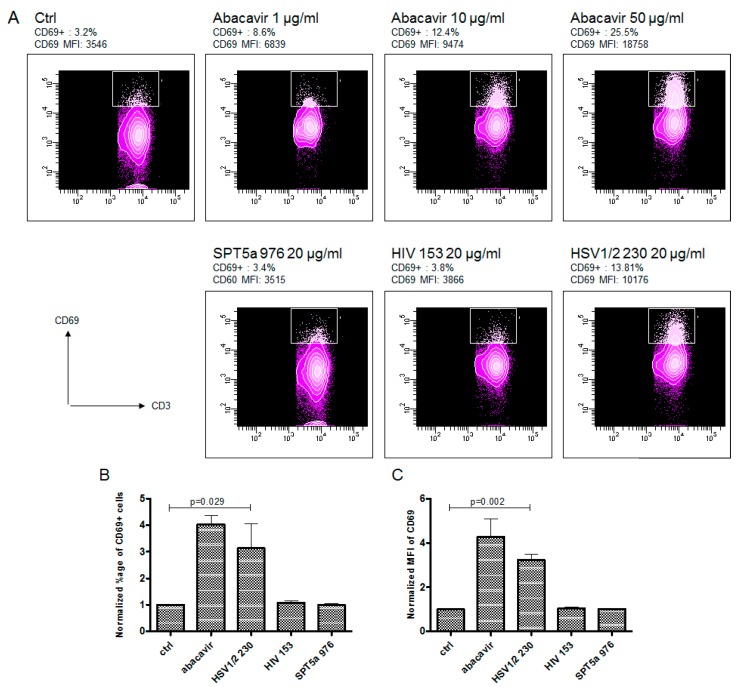
An abacavir-reactive TCR transfectant recognizes a virus peptide. An example of flow cytometric analysis (**A**) shows activation of JRT3-CD8 cells expressing a TCR derived from an abacavir-reactive T cell clone (BeS-B7) following stimulation with antigen presenting cells (721.221) expressing HLA-B*57:01 with increasing concentrations of abacavir (upper row) or with the different tested peptides (lower row). TCR transfectants show upregulation of the activation marker CD69 after stimulation with abacavir and VTQQAQVRL (HSV 230). The experiment was repeated 3 times; upregulation of CD69^+^ cells and mean fluorescence intensities of CD69 were normalized to control and are shown in (**B**,**C**), respectively. Ctrl indicates vehicle control (no peptide, no abacavir). Indicated *p*-values were calculated using the Student’s *t*-test.

**Figure 3:**
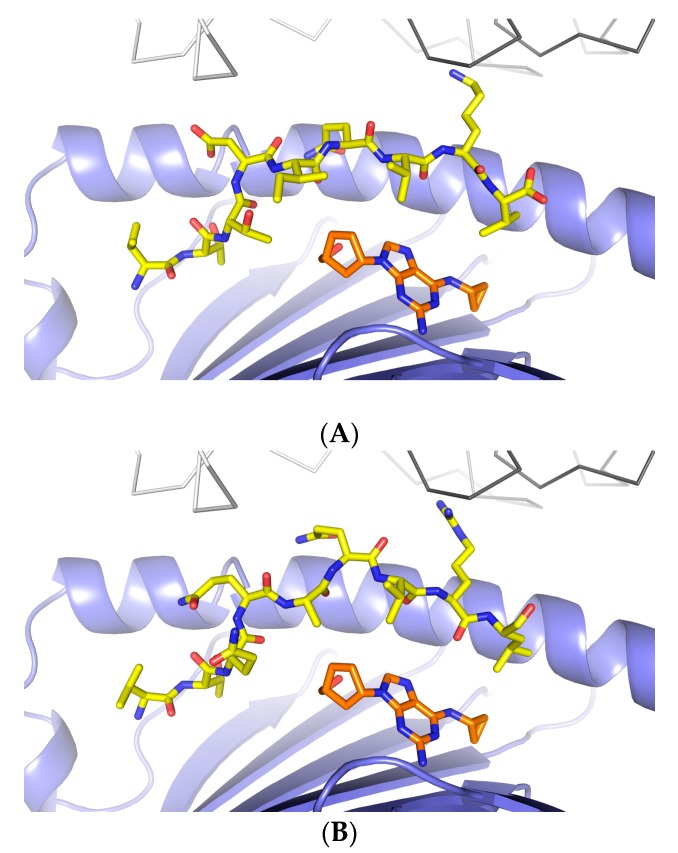
Structural similarity between self and viral peptides that bind abacavir in the context of HLA-B*57:01. (**A**) shows the crystal structure of the VTTDIQVKV SPT5a 976–984/abacavir/HLA-B*57:01 complex. (**B**) shows a model of VTQQAQVRL, corresponding to HSV1/2 230–238, complexed to abacavir and HLA-B*57:01. The HLA-B restricted TCR (gray lines) from 2NX5 was superimposed on HLA-B*57:01.

To determine if drug reactive T cells recognize viral peptides similar to VTTDIQVKV SPT5a 976–984, transfectants expressing TCRs derived from abacavir-specific T cell clones were challenged with the viral peptides listed in Table 1, as described previously [19]. The original T cell clones were derived from drug-naïve individuals by repeated stimulation with abacavir/autologous PBMC and obtained by limited dilution as described in [20]. Transfectants expressing 3 different TCRs (TCR BeS-B7, TCR 2D, TCR UL3L) from abacavir-stimulated clones were exposed to viral peptides and the homologous self peptide VTTDIQVKV SPT5a 976–984. Each TCR was derived from a T cell clone capable of responding to abacavir in the context of antigen presenting cells expressing HLA-B*57:01. Each of the 3 TCRs utilized distinct TCRα- and β-chain sequences. Two TCRs (2D and UL3L) out of the 3 tested were activated by abacavir but did not react to any of the tested peptides (Supplemental Figure S1). However, the TCR BeS-B7 transfectant could be activated by abacavir and also by VTQQAQVRL, corresponding to HSV1/2 230–238 (Figure 2). These data suggest that a subset of abacavir-specific T cells exhibit self/virus cross-reactivity when presented viral peptides that are homologous to drug-restricted self-epitopes. Structural modeling of VTQQAQVRL, HSV1/2 230–238, bound to abacavir and HLA-B*57:01, suggests that the most solvent accessible side chain, P8R, may be presented to the TCR in a manner consistent with how VTTDIQVKV SPT5a 976–984, P8K, may presented to the TCR (P8 K or R in close proximity to TCR α CDR3 (Figure 3).

## 4. Discussion

Drugs that elicit unwanted immune responses induce strong systemic or tissue-specific T cell responses [21,22]. The structural elements recognized by drug-induced T cells are not clear, but appear to be important factors driving pathogenic T cells specificity. Understanding the targets for T cell recognition are fundamental to comprehending mechanisms of drug hypersensitivity [23,24], and to improve prediction/prevention strategies [25].

In this study, we addressed the question of what motifs drug-specific T cells recognize, using the HLA-B*57:01 restricted response to abacavir as a model. An increasing number of associations have been found in recent years between specific HLA alleles and adverse drug reactions, including SJS/TEN, DRESS and DILI [1]. The association between abacavir hypersensitivity syndrome and HLA-B*57:01 [26,27,28] is the strongest HLA association among the known drug hypersensitivities, with OR = 960 [21]. The HLA restriction of drug responsive T cells has been characterized for several drugs [29,30,31], but the structural composition of HLA complexes recognized by pathogenic T cells that initiate, or perpetuate, drug-induced T cell responses are not well defined. Autoreactive drug-induced T cell responses may be directed at complexes consisting of HLA molecules bound to self or viral peptides. Alternatively, T cells may recognize complexes consisting of the drug, bound to peptide and HLA [23], with the drugs or their metabolites binding peptides and/or HLA molecules by covalent (hapten) [24] or non-covalent interactions. The composition of the HLA complexes recognized in vivo are not clear for several reasons: (1) patient samples are rare (estimated at 1–2 per million each year for SJS), (2) there are technical challenges to cloning and expanding drug specific T cells from patients, and (3) identification of the activating antigenic complex is technically difficult for isolated T cell clones, in which TCR could be considered as an orphan receptor. There are similar challenges in defining the specificity of autoreactive T cells from autoimmunity patients.

Since a number of drug hypersensitivity reactions and HLA alleles occur in patients with viral infections, viruses have been proposed to stimulate specific T cells that crossreact with self epitopes, thus resulting in autoreactive drug-induced T cell responses (the heterologous immunity model similar to molecular mimicry mechanisms of autoimmunity) [1]. There are several possibilities for how specific anti-viral T cell responses influence drug hypersensitivity: (1) viral epitopes may bind HLA in the absence of drug, (2) viral epitopes may bind HLA in the presence of drug, or (3) subpopulations of TCRs may bind drug directly, altering their affinity for specific peptide/hla motifs. The degree to which virus-specific T cells crossreact with self peptides in a manner that contributes to pathogenic responses is not clear.

We asked which elements in a self peptide recognized by abacavir hypersensitivity patients were most critical for T cell recognition. Since previously studied peptides complexed to abacavir and HLA-B*57:01 did not correspond to natural epitopes, this is the first study of peptide elements recognized by T cells from drug hypersensitivity patients. We solved the crystal structure of VTTDIQVKV, corresponding to transcription elongation factor SPT5 isoform A 976–984, complexed to abacavir and HLA-B*57:01 (Figure 1). The structure showed that the self peptide bound in a canonical manner with the expected exception of P9. HLA-B*57:01 exhibited a preference for bulky hydrophobic side chains in anchor residue P9, however, short aliphatic side chains, such as valine, were preferred at P9 in the presence of abacavir [15,32]. Since the conventional mode of TCR binding is contact between CDRs and solvent-accessible side chains of immunogenic peptide and HLA [33], we asked which peptide side chains were solvent accessible and most likely to be available for T cell recognition. The most solvent-exposed side chains in the VTT***D***IQV***K***V SPT5a 976–984 peptide were found at P4 and P8. The data suggested that a subset of drug-reactive TCRs contact self peptides in which P4D and P8K (or perhaps similar side chains at these positions) are presented in the context of HLA-B*57:01.

Since crossreactive mechanisms may influence abacavir hypersensitivity, we searched for viral peptides with sequence similarity to VTT***D***IQV***K***V SPT5a 976–984 (Table 1). The most similar viral sequences to VTT***D***IQV***K***V SPT5a 976–984 were derived from HIV and HSV. Based on sequence similarity, we expected virus sequences similar to VTT***D***IQV***K***V SPT5a 976–984 to bind HLA-B*57:01 in the presence of abacavir, potentially serving as targets for recognition by drug-reactive T cells.

There are challenges in generating sufficient numbers of drug-reactive T cell clones from patients to define peptide epitopes, there are alternative methods. We used TCR transfectants in which the TCR was derived from an abacavir reactive T cell clone (originally derived from PBMC of a normal HLA-B*57:01 individual). The abacavir reactive TCR was found to recognize VTQ***Q***AQV***R***L, corresponding to HSV1/2 230–238. Recognition of the response to this viral peptide was enhanced in the presence of abacavir. This pattern of recognition is similar to the response to the self peptide VTT***D***IQV***K***V SPT5a 976–984: partial recognition in the absence of drug, enhanced recognition in the presence of drug. Nevertheless, the TCR transfectants can be activated in the absence of this peptide, as long as abacavir is present. These data suggest that endogenous self peptides bind HLA-B*57:01 in the context of abacavir. Collectively, these data show that peptides of self or viral origin are capable of binding HLA-B*57:01 in the presence of abacavir and serving as targets for recognition by drug-reactive T cells.

Although there are challenges to studying relatively rare but extremely severe drug reactions, understanding biological mechanisms and genetic risk factors, such as HLA allele identity, has enormous potential in preventing drug hypersensitivity. For example, abacavir hypersensitivity syndrome is prevented by screening patients for HLA-B*57:01 [34]. The mechanism for abacavir hypersensitivity involves direct binding of the drug to the very strongly associated HLA molecule HLA-B*57:01 [15,32]. Associations between HLA alleles and many other drug hypersensitivities are strong (such as for carbamazepine and allopurinol) [1], but not as strong as for abacavir. These data suggest that mechanisms driving T cell responses to other drugs may be more complex than abacavir, involving multiple HLA molecules and/or additional factors [22]. Drugs may induce expression of proteins that bind associated HLA molecules. This study provides a framework for understanding structural elements recognized by drug induced T cells.

## Figures and Tables

**Table 1:** Virus sequences bear homology to a self peptide recognized by CD8^+^ cells from hypersensitivity patients.

Sequence	Protein	Sequence Number	Identity to SPT5a
VTTDIQVKV	Human SPT5A	976–984	100 %
VTTNIQTKV	HIV-1	153–161	77.8%
VTQQAQVRL	HSV1/2	230–238	44.4%
VTTDSVRAL	HSV1	12–20	44.4%

## Supplementary Materials

Supplementary materials can be found at www.mdpi.com/1422-0067/18/7/1464/s1.
