# Supplementary material for: Structural Elements Recognized by Abacavir-Induced T Cells
Source: Int J Mol Sci. 2017 Jul 7;18(7):1464. doi: 10.3390/ijms18071464 (PMC5535955; doi:10.3390/ijms18071464)
Supplement: Supplementary file 1 [file ijms-18-01464-s001.zip › ijms-196175-supplementary.pdf]

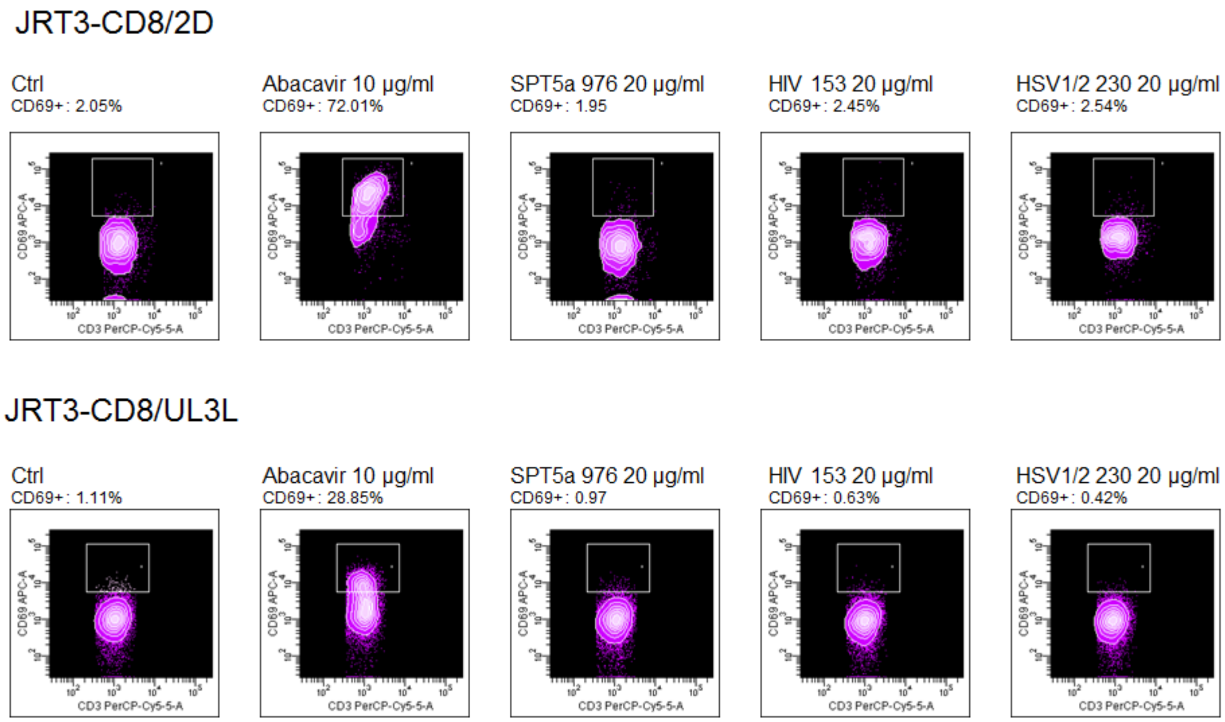

JRT3-CD8 cells expressing TCR 2D (upper pannel) and TCR UL3L (lower panel) were stimulated with abacavir or with peptides in the presence of 721.221 cells expressing HLA-b*57:01. After 16 hours of incubation, activation was monitored by CD69 upregulation using flow cytometry. JRT3-CD8 cells expressing TCR 2D or UL3L could be activated by abacavir but not by the tested peptides. Percentages indicate the proportion of events expressing CD69.
